# Effect of Alternating Current on the Cathodic Protection and Interface Structure of X80 Steel

**DOI:** 10.3390/ma10080851

**Published:** 2017-07-25

**Authors:** Huiru Wang, Cuiwei Du, Zhiyong Liu, Luntao Wang, De Ding

**Affiliations:** 1 Corrosion & Protection Center, University of Science & Technology Beijing, Beijing 100083, China; huiruwangustb@163.com (H.W.); liuzhiyong7804@126.com (Z.L.); ltwangustb@163.com (L.W.); 2 Shanxi Electric Power Research Institute, State Grid Corporation of China, Xi’an 710054, China; dingde5160@163.com

**Keywords:** AC corrosion, cathodic protection, neutral, alkaline, passivation, capacitance

## Abstract

This study employs potential-monitoring techniques, cyclic voltammetry tests, alternating current (AC) voltammetry methods, and surface characterization to investigate the AC corrosion of cathodically protected X80 pipeline steel. In a non-passive neutral solution at pH 7.2, a sufficiently negative potential completely protects steel at an AC current density of 100 A/m^2^. In an alkaline solution at pH 9.6, more serious AC corrosion occurs at more negative cathodic protection (CP) potential, whereas without CP the steel suffers negligible corrosion. In addition, the interface capacitance increases with AC amplitude. Based on these results, the AC corrosion mechanisms that function under various conditions are analyzed and described.

## 1. Introduction

Buried pipelines used for the transport of natural oil/gas are commonly protected from external corrosion by a cathodic protection (CP) system and organic coatings [1]. However, when pipelines are parallel to or crossing railways with alternating current (AC) electrification and high voltage transmission lines [2], AC interference can occur at coating defects or holidays and the corrosion rate of buried pipelines can accelerate, even if the pipelines are protected by CP with normal criteria (i.e., less than −850 mV (Cu/CuSO_4_ electrode, CSE) or 100 mV cathodic polarization) [3]. Therefore, AC corrosion of pipeline under CP protection requires urgent investigation.

In order to better understand the protective effects of different CP levels on AC corrosion, extensive work has been conducted based on numerous field tests and laboratory researches. Unfortunately, many different results have been obtained and the mechanism of AC corrosion under CP has not reached a consensus so far. Du et al. [4] proved that AC corrosion can be controlled by the increase of CP current when the AC current density is below 300 A/m^2^. Similarly, Cheng et al. [5,6] illustrated that only when the CP potential is sufficiently negative, will the steel be under a complete cathodic protection even when the AC current density is up to 400 A/m^2^. However, the experiments in these studies were conducted in relatively low-pH soil. Contrary phenomena have been observed in some other research works conducted in high-pH environments. Buchler et al. [7] demonstrated that in a alkaline soil solution, under high CP current densities, high corrosion rates may occur even when the AC voltage is low. They proposed that in the alkaline environment created by CP, an untouched passive film will be formed. AC corrosion can be avoided under low CP. There is more evidence for this alkalization mechanism. For example, Nielsen et al. [8,9], proposed that high dose CP is needed to prevent direct current (DC) interference and low CP can effectively protect steel from AC corrosion. In addition, a four-month field test conducted by Ormellese et al. [10] concluded that overprotection is the most dangerous condition for AC corrosion.

In addition, mathematical methods have been applied to illustrate the AC corrosion mechanism. The mathematical models developed by Lalvani [11], Ibrahim [12], and Bosch [13] confirm the existence of higher harmonic components of the faradaic current response when the AC amplitude is large. In addition, Lazzari [14] and Wang et al. [15] used the harmonic and fundamental current observed via AC voltammetry [16,17] to explore the nature of the electrode process. Specifically, the significance of soil resistance has been addressed. Lalvani et al. [11] developed a nonlinear mathematic model comprising solution resistance elements, to demonstrate that the AC corrosion rate can decline with increasing solution resistance. However, Buchler [7] proved that the acceleration of AC corrosion at high CP current densities cannot be primarily attributed to the high AC current densities caused by low spread resistance.

According to the different research results mentioned above, the debate about the influence of cathodic current on AC corrosion of pipelines is strongly connected to soil pH. Unfortunately, most published research was conducted in constant, unchanging environments, although progress on this issue required systematic comparison of AC corrosion mechanisms in different soil conditions. Thus, the present work investigated the AC corrosion of X80 pipeline steel under various CP potentials in two simulated soil solutions with different pHs.

## 2. Experimental

### 2.1. Electrodes and Solutions

The electrodes used in this work were cut from a sheet of X80 steel (chemical composition in Table 1). X80 steel cubes with dimensions of 10 mm × 10 mm × 4 mm were manufactured and embedded in epoxy resin, leaving a 10 × 10 mm^2^ working area. The specimens were carefully prepared to avoid bubbles and gaps at the metal/epoxy resin interface. The working surface of each electrode was gradually ground with 240 to 2000 grit sandpaper, rinsed with deionized water and methanol, and finally dried in air.

A high pH carbonate/bicarbonate solution and a neutral pH bicarbonate solution were used to simulate two typical service environments, pipelines are encountered in services, i.e., the CP effective trapped electrolyte under coating holidays and the soil electrolyte adjacent to the pipeline steel. The high pH carbonate/bicarbonate solution contained 0.05 M Na_2_CO_3_ and 0.1 M NaHCO_3_, which was open to air, with a pH of 9.6 and conductivity 15.2 mS/cm. The neutral pH solution contained 0.02 M NaHCO_3_ purging with 5% CO_2_/N_2_ mixture gas, with a solution pH of 7.2 and conductivity 2.6 mS/cm. All solutions were prepared from analytic grade reagents (Fisher Scientific, Shanghai, China) and ultra-pure water (18 MΩ·cm resistivity). The coated samples will be investigated in a following paper.

All tests were conducted at room temperature of 23 °C.

### 2.2. Immersion Tests

**Figure 1:**
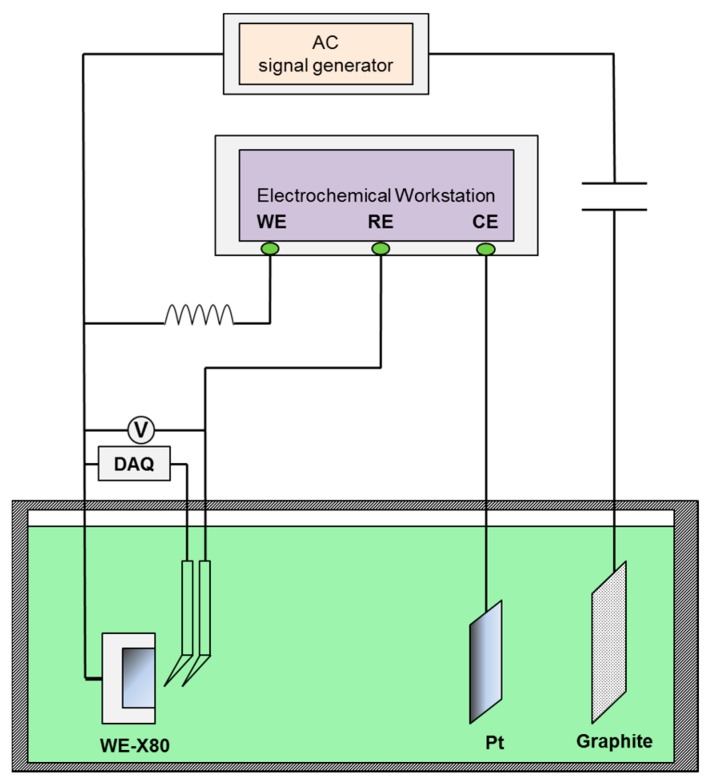
Experimental set up for direct current (DC) potential and real time potential measurements of X80 steel under various alternating current (AC) densities and galvanostatically applied cathodic protection (CP).

The X80 steel specimens were immersed in the two experimental solutions for 120 h, with each specimen exposed to a different AC current density (0, 30, and 100/500 A/m^2^), and to various CP potentials (i.e., zero, a minimum CP potential (100 mV cathodic polarization), −1000 mV (saturated calomel electrode, SCE), and −1200 mV_SCE_). The CP potentials were the resulting potentials of galvanostatic polarization [5], and pre-determined constant cathodic current values were used as the parameters. A 50 Hz sine-wave AC current was applied, with the AC current density defined as the ratio of the root mean square (RMS) current to the specimen area. The RMS current was measured using a Victor 86e multimeter (Victor, Shenzhen, China). The experimental setup was similar to that used for the DC potential measurements (Figure 1 in Section 2.3), except that the data acquisition (DAQ) device used to record the real-time potential was removed during the immersion test.

After immersion testing, the morphology of the corrosion products of each specimen was acquired by using an optical microscope. Confocal Raman spectroscopy was conducted to further characterize the corrosion product films in pH 9.6 solutions. The type of laser was a He-Ne laser of 785 nm, and the laser power was 5 mW. The lens magnification was 50× with a 2 um laser facula. The scan range was 100–2000 cm^−1^, and the acquisition was repeated three times. Then the expoxy resin was removed, and a solution of 500 mL HCl, 3.5 g hexamethylenetetramine, and 500 mL distilled water was used to remove the corrosion products deposited on the specimen’s surface according to ASTM G1-81 [18]. The cleaning process lasted for 10 min and repeated on specimens for three times to make sure that the corrosion products had been removed thoroughly. The weight loss of the steel specimens was measured using an electronic balance with an accuracy of 0.1 mg. After cleaning, the surface morphology was analyzed by a Quanta250 environment scanning electron microscope (SEM) (Advanced Microscopy Laboratory, LMA, Zaragoza, Spain).

### 2.3. DC and Real-Time Potential Measurement

Under various CP potentials and AC current densities, DC potential and real-time AC/DC potential measurements of the X80 steel were performed in pH 7.2 and pH 9.6 solutions to investigate interactions between CP and AC on X80 steel in non-passive and passive systems. Figure 1 shows the experimental set up in which a four-electrode system was used to apply constant CP current. The current parameters were pre-determined to obtain the required CP potentials. The X80 steel was used as the working electrode (WE), a platinum sheet served as the counter electrode (CE) and two saturated calomel electrodes (SCE) served as independent reference electrodes (RE) to avoid mutual interference between the data-acquisition devices and the electrochemical workstation (DC source). The potential between the two reference electrodes was 1 mV or less. The ATF05C DDS (Saelig Company, New York, NY, USA) function waveform generator, the coupon, and graphite electrode, constituted the AC circuit. In addition, a capacitor installed between the specimen and the AC signal generator was used to prevent DC from flowing into the AC circuit, and an inductor placed between the electrochemical workstation and the specimen prevented AC from entering the DC source. The DC potential of X80 steel specimens was measured using a Victor 86e multimeter. After the DC potential reached steady state, the real-time AC/DC potential was recorded using a National Instruments USB-6351 multifunction (DAQ) card (National Instruments Company, Shanghai, China) with 1 kHz acquisition frequency.

Electrochemical impedance spectroscopy (EIS) tests were conducted for two different solutions after the samples were stabilized in the open circuit potential (OCP), stabilized for 30 min. The EIS was obtained in the 100 kHz to 0.01 Hz frequency range with six points per decade and 10 mV perturbation amplitude of 10 mV. Potentiodynamic polarization measurements were conducted on the specimen with a 0.5 mV/s potential sweep rate in a potential range from −1.2 to −0.4 V_SCE_ (pH 7.2) and from −1.2 to 2 V_SCE_ (pH 9.6).

### 2.4. Cyclic Voltammetry Measurements

The cyclic-voltammetry (CV) technique was used in the pH 9.6 alkaline environments to simulate and further investigate the AC corrosion process with the existence of passive film. A traditional three-electrode system was used with a Versa studio 3 electrochemical workstation (AMETEK, Beijing, China). Before cyclic voltammetry measurement, open circuit potential (OCP) tests were conducted before and after cathodic polarization. Cathodic polarization was applied to the electrode surface at −1 V (SCE) for 1200 s after 1 h immersion, then the cathodic current was stopped and the sample was immersed for 1 h. The change in open circuit potential (OCP), before and after cathodic polarization, was recorded to illustrate the passive film properties. During the CV test, to simulate AC with different amplitudes, the potential E swept linearly from −1.2 to 0.3 V_SCE_ and −1.8 to 1.5 V_SCE_. To better investigate the electrode process mechanisms and due to the equipment limitations, a medium scan rate of 50 mV/s was used to simulate low frequency AC [19]. The frequencies calculated, based on scan rate and scan range, were 16.7 mHz (−1.2 to 0.3 V_SCE_) and 7.58 mHz (−1.8 to 1.5 V_SCE_).

### 2.5. AC Voltammetry Technique

The working electrodes were subjected to AC voltammetry measurements (Mott–Schottky tests in VersaSTAT 3, (AMETEK, Beijing, China) with a DC potential ranging from −1.2 to 1.5 V_SCE_ in pH 9.6 alkaline solutions to investigate the metal/solution interface properties. A traditional three-electrode system was applied. AC voltage signals (50 Hz) at constant amplitude (100, 200, 400, 600, 800, and 1000 mV (root mean square value)) were imposed on the X80 steel specimens to characterize the influence of AC on the interface capacitance.

From the perspective of instrumentation process, the AC voltammetry technique is appropriate for capacitance measurement when large amplitude AC is applied. In the AC voltammetry technique, the current response may be recorded using the data-acquisition equipment from the output terminal of an electrochemical workstation for further harmonic analysis (this will be discussed in future work), or it may be directly processed by the electrochemical workstation to acquire impedance information. The large-amplitude voltammetry technique used here constitutes the latter approach in which a discrete Fourier transform (DFT) is performed to decompose the stored and averaged image from many periods into frequency components, and the impedance is then calculated as the quotient of the fundamental share of excitation potential and current response signals.

Because the current-potential relationship of the corroding system has a nonlinear nature [13], for large amplitude AC voltammetry the frequency content of a faradaic current component is not limited to ω (the frequency of excitation potential), and numerous higher harmonics can be generated. This is different from traditional AC voltammetry with small excitation signals such as EIS [15]. Therefore, the instrumentation process described above cannot be applied to calculate faradic resistances when a large amplitude AC voltage signal is added, because only fundamental signals are used in the calculation, whereas copious higher harmonic signals containing useful faradaic information are abandoned. However, measuring capacitance through this instrumentation process is practical because the fundamental signal is sufficient to estimate the capacitance. The faradaic element acts as a harmonic current generator, meaning that the capacitance effect is minimized in the second harmonic, and the fundamental harmonic signal has a high capacitance to faradic current ratio [16,19]. The capacitance can be derived from the imaginary part of the impedance. Based on several assumptions that are discussed later in Section 3.5, the capacitance can be calculated using the Equation (1) [15].

(1) $$C\;=\;-\frac{1}{\omega Z^{\prime \prime }}$$

where Z″ is the imaginary component of the interfacial impedance and, ω is the angular frequency.

## 3. Results and Discussion

### 3.1. Weight Loss Measurement and Surface Morphology Characterization

**Figure 2:**
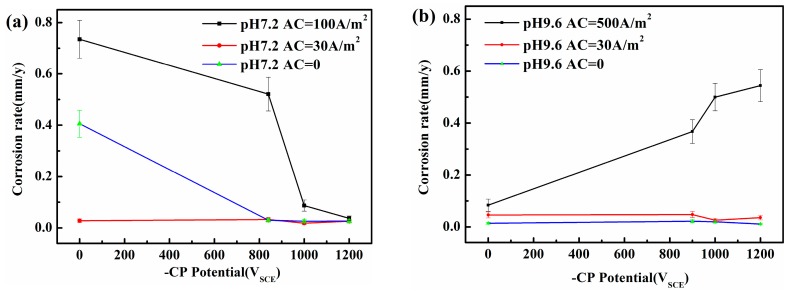
Corrosion rate of X80 steel under various i_AC_ and CP potentials after 120 h immersion in simulated soil solutions with two different pHs: (**a**) pH 7.2; (**b**) pH 9.6.

Figure 2 shows that in the absence of AC or when i_AC_ is small (i.e., 30 A/m^2^), the steel may be protected from corrosion by the passive film [20,21,22,23] in the pH 9.6 solution with or without CP. However, in the neutral solution, serious corrosion occurs when both CP and AC are absent, whereas corrosion may be negligible with i_AC_ 30 A/m^2^. In the neutral pH 7.2 environment, corrosion can occur at open circuit potential and the acidification under the corrosion product layer will be caused by HCO_3_^−^, increasing the corrosion rate. The cathodic polarization in the negative cycle of AC increases the local alkalinity and reduces corrosion rate. Increasing the local alkalinity can promote the accumulation of corrosion products. When the AC is relatively small (30 A/m^2^), this corrosion product film can block the ions released by anodic dissolution [20], decreasing the corrosion rate. When AC is large, this fragile corrosion product film in the neutral solution can be easily attacked and destroyed, and serious corrosion can occur. When the AC amplitude is large, the AC corrosion in the neutral solution can be under controlled in the presence of a more negative CP potential (−1000 and −1200 mV_SCE_) when i_AC_ is up to 100 A/m^2^. However, in the alkaline solutions, and especially in the pH 9.6 solution, the AC corrosion rates strongly increase with the CP level.

**Figure 3:**
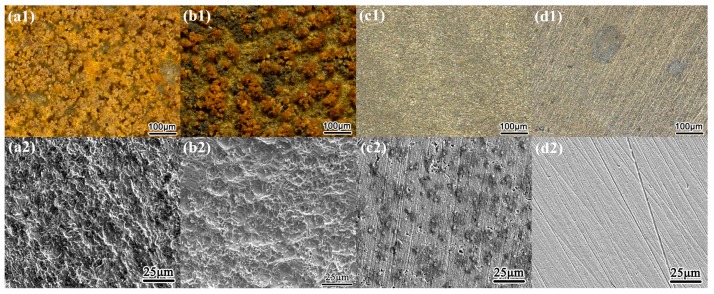
Surface morphology before and after removing the corrosion products of X80 steel after 120 h of exposure to pH 7.2 solutions with the application of 100 A/m^2^ AC under various CP: (**a1**) 0 mV_SCE_, with products; (**b1**) −840 mV_SCE_, with products; (**c1**) −1000 mV_SCE_, with products; (**d1**) −1200 mV_SCE_, with products; (**a2**) 0 mV_SCE_, without products; (**b2**) −840 mV_SCE_, without products; (**c2**) −1000 mV_SCE_, without products; (**d2**) −1200 mV_SCE_, without products.

**Figure 4:**
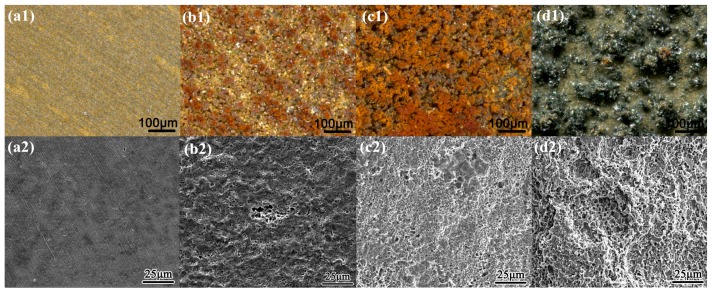
Surface morphology before and after removing the corrosion products of X80 steel after 120 h exposure to pH 9.6 solutions with the application of 500 A/m^2^ AC under various CP: (**a1**) 0 mV_SCE_, with products; (**b1**) −900 mV_SCE_, with products; (**c1**) −1000 mV_SCE_, with products; (**d1**) −1200 mV_SCE_, with products; (**a2**) 0 mV_SCE_, without products; (**b2**) −900 mV_SCE_, without products; (**c2**) −1000 mV_SCE_, without products; (**d2**) −1200 mV_SCE_, without products.

Figure 3 and Figure 4 present the surface morphology of X80 steel specimens before and after removing the corrosion products after 120 h of exposure to relatively large amplitude AC with various CP levels in the two different environments. In the neutral pH 7.2 solution, when there was no cathodic protection or at traditional −840 mV cathodic protection potential, the AC corrosion was serious. At more negative cathodic potential, such as −1000 mV_SCE_ or −1200 mV_SCE_, the AC corrosion was mild. In the pH 9.6 solutions, a porous corrosion product film deposited on the surface in the presence of CP under AC, and the corrosion accelerated as the CP level increased, as shown in the corrosion products morphology results in Figure 4(b1–d1). Few corrosion products can be observed when CP is absent (Figure 4(a1)), and the surface is protected relatively well (Figure 4(a2)). Measured corrosion rates imply that the influence of AC interference on the X80 steel under CP depends on pH conditions, which is especially distinct in non-passive and passive systems. To further illustrate their respective mechanisms, we measured the DC and real-time potentials in pH 7.2 and pH 9.6 solutions.

### 3.2. Electrochemical Measurements

#### 3.2.1. Analysis of DC Potential

**Figure 5:**
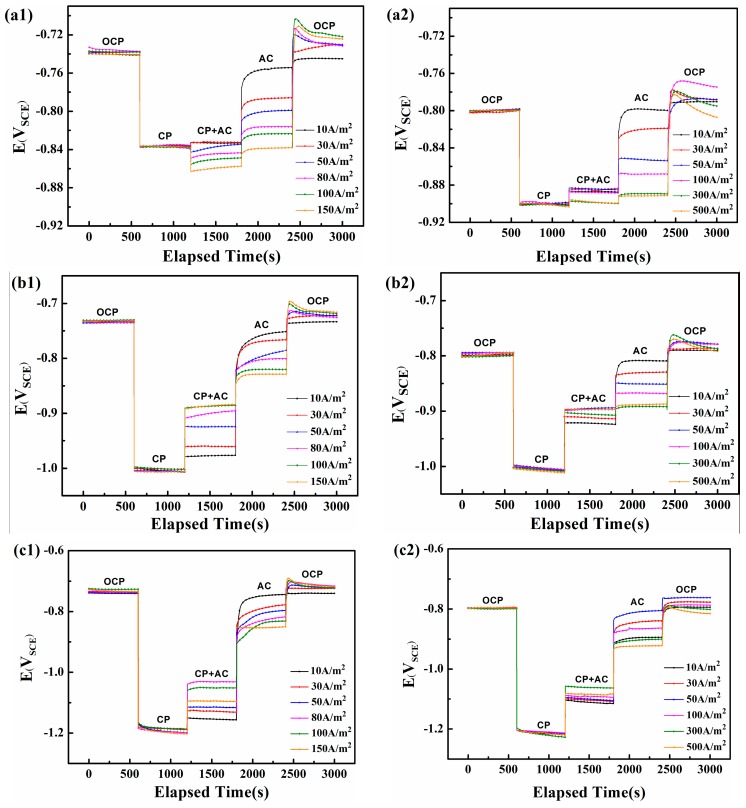
The DC potential recorded on the steel electrode under various i_AC_ and different CP in two soil solutions with pH 7.2 and pH 9.6: (**a1**) CP = −840 mV_SCE_, pH 7.2; (**b1**) CP = −1000 mV_SCE_, pH 7.2; (**c1**) CP = −1200 mV_SCE_, pH 7.2; (**a2**) CP = −900 mV_SCE_, pH 9.6; (**b2**) CP = −1000 mV_SCE_, pH 9.6; (**c2**) CP = −1200 mV_SCE_, pH 9.6.

Figure 5(a1–c1) shows the variations of surface DC potential of steel in neutral solution (pH 7.2). Based on the National Association of Corrosion Engineers (NACE) standard [24], when a minimum CP potential of −775 mV_SCE_ cannot protect a pipeline from corrosion, a minimum of 100 mV cathodic polarization should be applied. Consequently, the normal CP potential of steel in neutral and alkaline environments is −840 and −900 mV_SCE_, respectively, which corresponds to their corrosion potential (−740 and −800 mV_SCE_) obtained from 0 to 600 s in Figure 5. The galvanostatic polarization technique is applied on the steel surface from 600 to 1200 s to obtain the required CP potential. Next, alternating currents with various current densities are applied from 1200 to 1800 s, following which the CP is stopped from 1800 to 2400 s. Finally, both the AC and CP are ceased from 2400 to 3000 s.

Figure 5(a1) shows the change in DC potential for various AC current densities under a −840 mV_SCE_ CP potential. All DC potentials attain a steady state at −840 mV_SCE_ after the galvanostatic polarization cathodic current is applied. The influx of AC above 30 A/m^2^ shifts the DC potential downward, with the decline increasing with AC density. Only when the AC interference is relatively small (below 30 A/m^2^), will the DC potential increase slightly (from −840 mV_SCE_ to −830 mV_SCE_). When the CP is stopped at 1800 s, the DC potential shifts sharply upward and then gradually increases until it reaches a steady state. The steady state DC potential becomes more negative as the AC density increases. Finally, when the CP and AC are both stopped at 2400 s, the DC potential immediately surges to a positive peak and then gradually decreases to the crossover potential (defined at the zero-overall DC current).

When the CP potential is −1000 mV_SCE_ or −1200 mV_SCE_ (Figure 5(b1,c1)), the DC potential first remains at −1000 or −1200 mV_SCE_ and then shifts upward when the AC is applied until it reaches a steady state. The steady-state DC becomes more positive with increasing AC density until 100 A/m^2^, following which it shifts upward at i_AC_ = 150 A/m^2^. With no CP, the DC potential is again positively shifted and the new DC potential becomes more negative with increasing i_AC_.

The trends in the DC potential reverse when the working electrode is under low or high CP. Under the traditional CP of −840 mV_SCE_, the DC potential shifts negatively, but a positive shift occurs for CP of −1000 and −1200 mV_SCE_. Lillard et al. proved that a net DC polarization would be induced by AC current [25,26], and the corrosion rate under 60 Hz AC generally agreed with the average AC current [25]. The shift of crossover potential under AC can be attributed to asymmetry in the anodic and cathodic Tafel slopes (β_a_ and β_c_) [27,28,29,30,31] and the reaction with the lower Tafel slope may preferentially accelerate with the application of AC potential [26,31]. However, few mechanisms have been proposed for the influence of AC on the CP potential. Du et al. [4,32] suggested that the shifting direction of the CP potential might depend on the varying slopes of the three parts of the cathodic polarization curve, i.e., the activation control stage with low slope, the oxygen diffusion control stage with high slope, and the mixed control stage with low slope. This theory was limited to diffusion-controlled systems, and neglects the effect of anodic reaction. Because the 0.02 M NaHCO_3_ solution is aerated, the cathodic polarization process is completely controlled by electrochemical activation, which means that electron-transfer on the specimen surface plays a significant role in determining the DC potential and needs to be discussed in detail.

The currents that participate in the electrode process can be divided into two types: electron suppliers and electron consumers. The anodic reaction current density i_a_, the cathodic protection current i_cp_, and the AC negative cycle current density i_AC_^−^ can be categorized as electron suppliers, and the cathodic reaction current i_c_, and the AC positive cycle current density i_AC_^+^ can be categorized as electrons consumers. The i_AC_^+^ and i_AC_^−^ are the faradaic part of AC, because the capacitive currents of the negative cycle and positive cycle sum to zero [25].

Under a minimum CP of −840 mV_SCE_, i_cp_ is relatively small. The electrons provided by the cathodic protection current (i_cp_) are relatively few, therefore the electrons consumed in the AC positive cycle (i_AC_^+^) should be provided by the dissolution of iron (i_a_) and AC corrosion is induced. Meanwhile, the anodic and cathodic polarization during an AC positive and negative cycle is not symmetrical [6,13], and the residual electrons provided by the AC negative cycle (i_AC_^−^) can shift the potential negatively. This faradaic process serves as a rectifier [33,34,35] and a net cathodic DC current is induced after the AC flows through. Cheng et al. [5] studied a similar phenomenon and concluded that the negative shift of the potential proves that the anodic dissolution is accelerated and provides excessive electrons. However, they neglected to consider that the increase in free electrons could also be due to the negative cycle of the externally applied AC and the anodic dissolution is driven by the positive cycle of the externally applied AC (i_AC_^+^).

When the CP is −1000 or −1200 mV_SCE_, i_cp_ is relatively large. The cathodic protection current (i_cp_) provides enough electrons to be consumed by the AC positive cycle (i_AC_^+^), so corrosion will not be triggered. As part of the cathodic protection current (i_cp_) is consumed by i_AC_^+^, the original negative potential cannot be maintained, and will be shifted positively.

Figure 5(a2–c2) shows how the AC influences the electrode potential in the alkaline solution (pH 9.6). The application of AC increases the DC potential for all three initial CP potentials (−900, −1000, and −1200 mV_SCE_), which means that AC corrosion should be controlled at these three CP levels if the corrosion mechanism in this pH 9.6 solution is similar to that in the pH 7.2 solution. However, according to the weight loss measurements shown in Figure 2, the corrosion rates in the pH 9.6 condition gradually increase with CP level and exceed 0.1 mm/y. In addition, the DC potential for CP at −900 mV_SCE_ and −1000 mV_SCE_ increases with AC current density up to 100 A/m^2^, after which their positive shift is less marked, from i_AC_ = 100 A/m^2^ to 500 A/m^2^, whereas the turning point for CP of −1200 mV_SCE_ is 300 A/m^2^. These results differ from those obtained for the neutral solution, which may be due to the passivation characteristics of X80 steel that block the free transfer of electrons and ions [36].

#### 3.2.2. Real Time Potential Measurement

**Figure 6:**
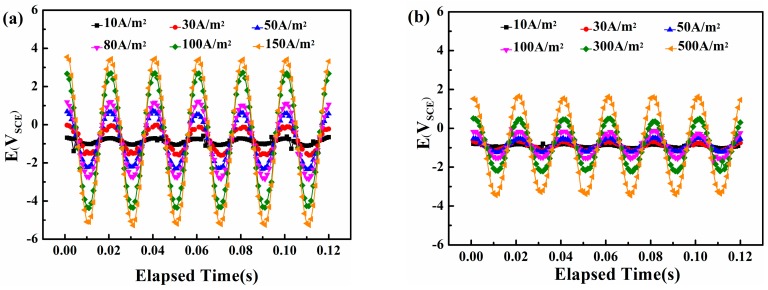
Real-time AC/DC potential waveform records of X80 steel under traditional CP potential and various AC densities in two solutions: (**a**) pH = 7.2, CP = −840 mV_SCE_; (**b**) pH = 9.6, CP = −900 mV_SCE_.

Figure 6 shows the real-time potential recorded by the data-acquisition system on the steel surface in neutral and alkaline solutions A 50 Hz sine wave appears, which means that the original AC interference signal passes into the steel surface without distortion. The amplitude of the real-time potential grows with the increase in AC density.

**Figure 7:**
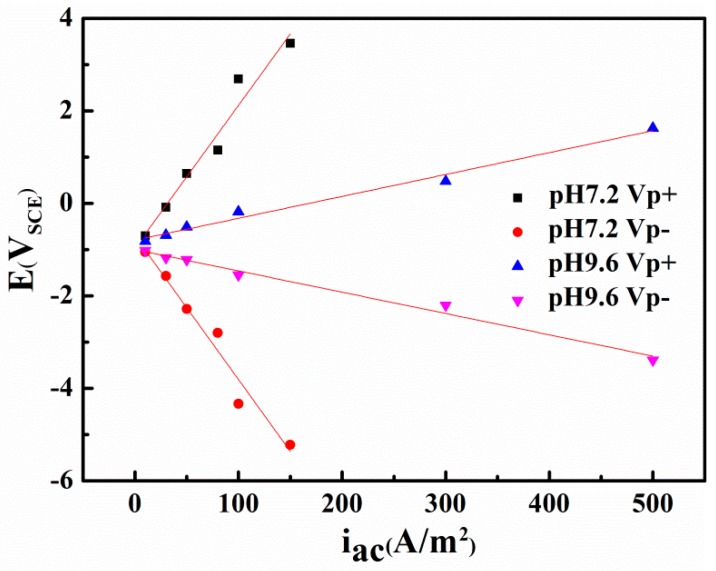
The most positive potential (VP^+^) and the most negative potential (VP^−^) as a function of AC current density (i_AC_) in two solutions with pH 7.2 and pH 9.6.

Figure 7 compares the peak and valley real-time potential of the steel in neutral and alkaline environments. A clear linear correlation appears between the AC current densities and the two characteristic voltages (i.e., the Vp^+^ and Vp^−^) in neutral and alkaline conditions. However, the most positive potential and the most negative potential are 3.46 and −5.22 V_SCE_, respectively, in the neutral solution with an AC current density of 150 A/m^2^, whereas in alkaline environments, the extremes are only 1.63 and −3.39 V_SCE_ even when the AC current density is 500 A/m^2^. This distinction can be attributed to the conductivity difference of the two solutions, and indicates that using the critical AC current density or AC voltage to evaluate corrosion can lead to different results.

#### 3.2.3. EIS and Potentiodynamic Polarization Studies

**Figure 8:**
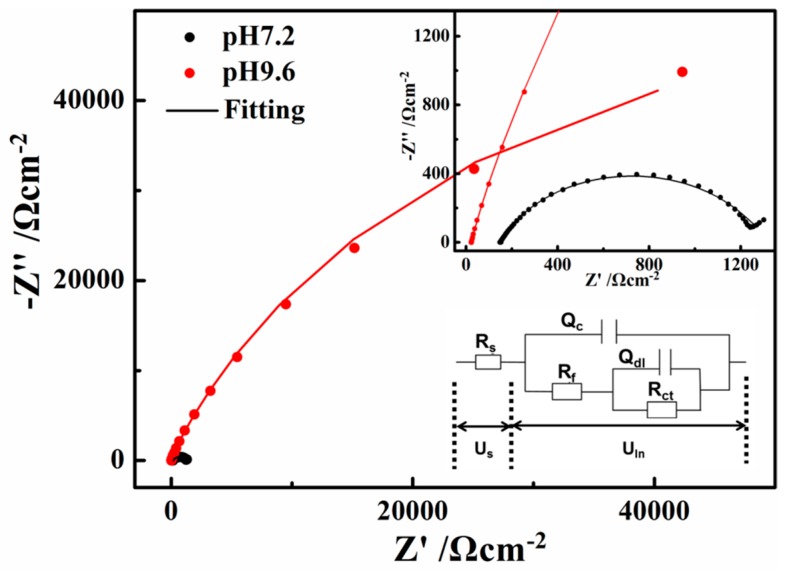
Electrochemical impedance spectroscopy (EIS) results of X80 steel in the pH 7.2 and 9.6 solutions: Nyquist plots.

The open circuit impedances of X80 steel in the two solutions are presented in Figure 8 as a Nyquist diagram. The radius of the semi-circular arc is related to the polarization resistance of the passive film [37]. Therefore, the measured impedance was large in the pH 9.6 solution, while the semi-circles appeared to be more depressed in the pH 7.2 solution, indicating that the corrosion resistance ability is the weakest in the pH 7.2 solution.

The two R–Q elements equivalent circuit in Figure 8a was adopted to fit the experimental data according to certain references [38,39,40,41]. The solution resistance (R_s_) in the pH 7.2 solution (149.6 Ω·cm^−2^, according to the fitting results) is much larger than that in the pH 9.6 solution (22.63 Ω·cm^−2^), which greatly contributes to the differences in the current-voltage relationship in the neutral and alkaline solutions. R_ct_ reflects the difficulty of charge transfer and the corrosion resistance ability of the steel. R_f_ represents a resistance due to the ionic paths through the oxide film, parallel with Q_c_ which represents the capacitive behavior of the passive film. Q_c_ decreased from pH 7.2 (2.458 × 10^−4^ Ω^−1^·cm^−2^S^−n^) to pH 9.6 (9.14 × 10^−5^ Ω^−1^·cm^−2^S^−n^), indicating that the defects on the surface film decreased. Both the values of R_f_ and R_ct_ are much larger in the pH 9.6 alkaline solution (9196 and 125,300 Ω·cm^−2^) than that in the pH 7.2 solution (1153 and 845 Ω·cm^−2^), indicating that the passive film formed on the X80 steel surface in the pH 9.6 alkaline solutions has good protective abilities [35]. The transfer of ions and charge can be greatly inhibited by the films in the pH 9.6 alkaline solutions. This phenomenon is discussed in Section 3.3 to investigate deeply the influence of passive film on ions and charge transfer during AC cycles.

**Figure 9:**
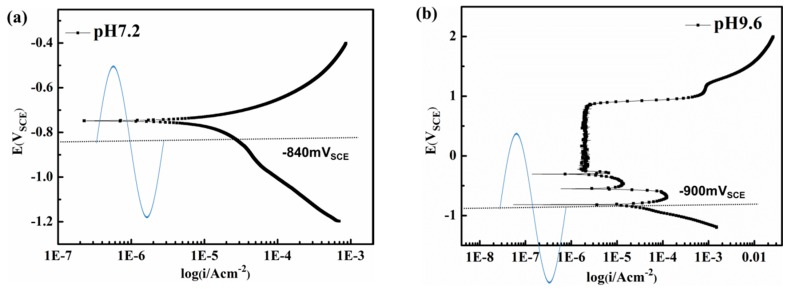
Potentiodynamic polarization curves. (**a**) pH 7.2 solution; (**b**) pH 9.6 solution.

To investigate the fluctuation influence of the real-time AC potential on corrosion of the metal, potentiodynamic polarization studies were conducted. As seen from Figure 9, with the application of AC and minimum CP, the real time potential fluctuates between passive, active, and corrosion immune region [19,42] in the pH 9.6 solutions, whereas in the pH 7.2 solution the sample experiences alternating anodic dissolution and cathodic reduction.

The results of potential measurement, EIS and potentiodynamic polarization indicate that the corrosion performance depends on the influence of AC on CP potential in the neutral solution. Unlike in neutral conditions, the corrosion rates in alkaline conditions cannot be attributed to the shifts in the CP potential brought by the AC. The passive film formed on the steel surface in the alkaline environments can inhibit ions and charge transfer processes, and the film forming-dissolution step during the potential fluctuation may play a significant role and require further consideration.

### 3.3. Short and Long-Range Cyclic Voltammetry Analysis

**Figure 10:**
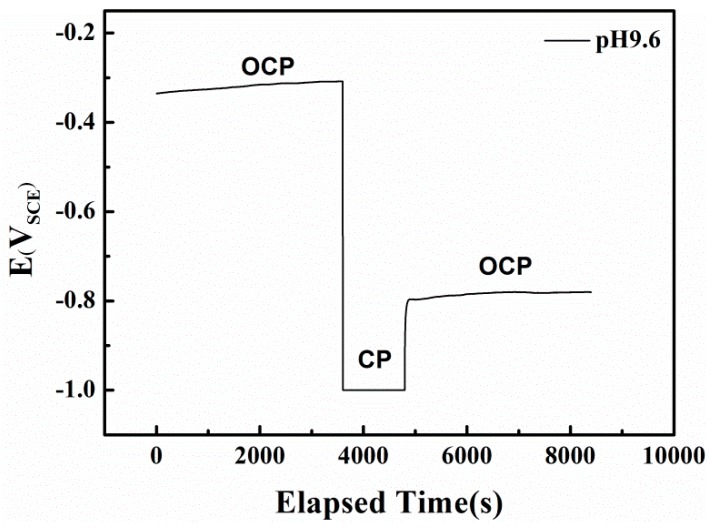
The influence of potentiostatically cathodic polarization on open circuit potential of X80 steel in pH 9.6 solution.

In the alkaline solution, the redox reactions of the specimen are controlled by both the electron-transfer and film forming-dissolution steps. Because the dominant active species and the electrode potential can strongly influence the types and rates of anodic reactions [43,44], the AC corrosion mechanism for X80 steel under CP is closely related to the high HCO_3_^−^ concentration in the pH 9.6 solution. To investigate the passive film properties in pH 9.6 solution, the OCPs before and after cathodic polarization (CP in Figure 10) were recorded.

In pH 9.6 alkaline environment, when AC is absent and the specimen is at the OCP, a metastable film mainly composed of FeCO_3_ appears on the surface through the reaction of a previously formed film in air with bicarbonate ions in the solution, via the reverse reaction of Equations (6) and (7) [45]. As shown in Figure 10, this passive film can be removed by prior cathodic polarization without recovery, shifting the electrode surface to the anodic dissolution state. A similar effect in which the cathodic current removes the passive film in HCO_3_^−^ solution under OCP was previously reported [46].

**Figure 11:**
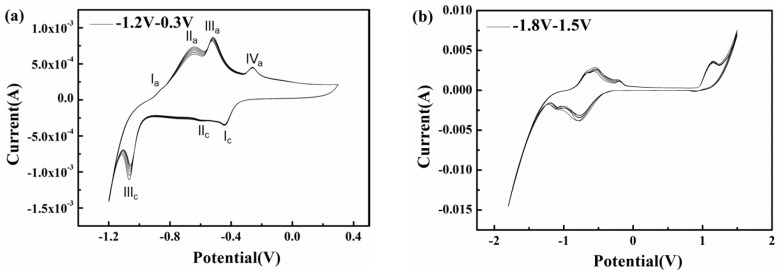
Cyclic voltammetry in slightly alkaline solutions with pH 9.6 at different ranges (**a**) −1.2 V_SCE_–0.3 V_SCE_; (**b**) −1.8 V_SCE_–1.5 V_SCE_.

To explore the influences of potential fluctuation on the corrosion characteristics of X80 steel in passivation systems, cyclic voltammetry experiments with short and long ranges were conducted in pH 9.6 alkaline environments (Figure 11). In the pH 9.6 solution, as shown in Figure 11a, a short range cyclic voltammetry from −1.2 to 0.3 V_SCE_ was conducted, and the positive vertex potential nearly corresponds to the most positive real time potential of 300 A/m^2^ AC (Figure 7). The scan rate of 50 mV/s can simulate an AC frequency of 16.7 mHz.

As the anodic scan progresses, the current rises sharply from −1.375 mA at −1.2 V_SCE_ to −231 μA at −1.06 V_SCE_ and steadily increases to zero at −937 mV_SCE_, as the hydrogen evolution reaction (HER) induced by water reduction controls the electrode process and increases the local alkalinity [47,48]. At a higher scan potential, the liberation of ferrous ions through anodic dissolution is gradually driven by the migration of OH^−^ and water molecules, resulting in the mitigation of cathodic predominance [49,50]. However, the electrode process remains under cathodic control, although the mechanism of the cathodic reaction transforms into the slower reduction of bicarbonate, producing carbonate and adsorbed hydrogen atoms [48,51]. As the scan progresses, the oxidation of iron occurs so that the reaction current becomes anodic and four peaks appear in the active domain. The first peak at approximately −869 mV_SCE_ may be related to the zero free charge potential of the X80 steel surface; it indicates the accelerated anodic dissolution. As the potential becomes more positive, the iron dissolution continues to accelerate while the hydroxyl and carbonate ions produced by cathodic reduction are absorbed and accumulate on the electrode surface, interacting with the ferrous ions released by the anodic reaction so that a layer of hydrated Fe(OH)_2_ precipitates onto the surface [46,52], resulting in the formation of peak IIa in Figure 11a. This initially formed monolayer of Fe(OH)_2_ is non-protective and can be easily attacked by the abundant bicarbonate on the interface [48,53,54,55,56]. Thus, this thin and fragile prepassive film dissolves and the bare metal substrate is again exposed. Nevertheless, carbonate ions produced during this process and the previous cathodic reduction of bicarbonate can combine with the released ferrous ions to gradually deposit a FeCO_3_ based passive film [57] that can develop sufficiently to spread over the surface and inhibit the further dissolution of metal, leading to the formation of peak IIIa in Figure 11a. A small peak (IVa) appears in the potential range in which the formation of ferric oxides is expected, which means only a small fraction of the FeCO_3_ is oxidized to Fe_2_O_3_. This is because the excessive bicarbonate ions inhibit the transformation of FeCO_3_. Beyond peak IV_a_, the curve exhibits a passive area with a current density as low as 212 μA/cm^2^ when the potential is greater than 100 mV_SCE_, indicating that a protective passive layer forms on the steel surface. In the reverse scan, three cathodic peaks with current densities that differ by orders of magnitude appear at −434 mV_SCE_, −598 mV_SCE_, and −1.06 V_SCE_. The reduction peaks (Peak Ic in Figure 11a) indicate the formation of Fe(OH)_2_ rust and the rust layers accumulate as the cycles increase(Peak IIIc in Figure 11a).

The corrosion induced by 16.7 mHz AC with a density of 300 A/m^2^ can be explained based on the CV results. As shown in Figure 11a, the passive film formed during the positive half cycle is metastable because it consists of a small portion of Fe_2_O_3_ (Peak Iva in Figure 11a) and a large portion of FeCO_3_. Under the low frequency (16.7 mHz) AC, the non-Faradic current approaches zero [25]. Therefore, the Faradic current induced by the cathodic reaction is large enough to reduce this unstable passive film and a porous rust layer mainly composed of Fe(OH)_2_ deposits on the X80 steel surface [58,59] (Peak Ic in Figure 11a). Further reduction of Fe^2+^ species [53,60] (Peak IIIc in Figure 11a) could be suppressed by abundant hydroxyl ions [61] due to limited solubility [7]. Fe(OH)_2_ rust gradually accumulates with AC cycles.

**Figure 12:**
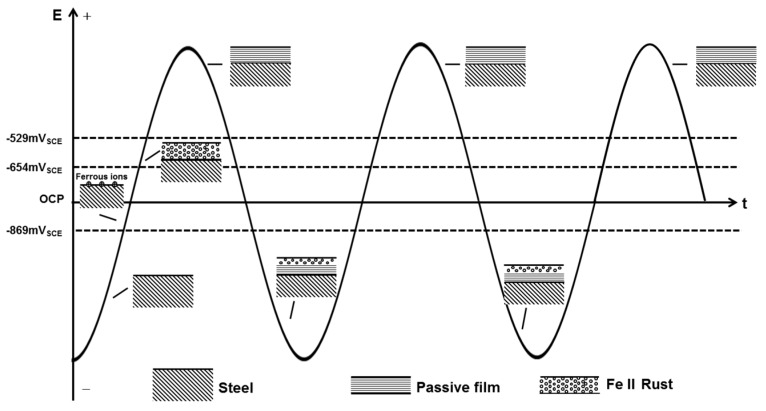
Sketch map of the AC corrosion process occurring on X80 steel in the absence of CP current.

However, in the presence of a practical 50 Hz AC, the charge-discharge process of the double layer consumes a majority of AC, leaving a small fraction to participate in the redox reaction of X80 steel in the alternating positive and negative half cycles [25,62]. In addition, as shown in Figure 10, the cathodic reduction potential can be as negative as −3.39 V_SCE_ (500 A/m^2^ AC). The reduction current is controlled primarily by the diffusion limited oxygen current density [25]. Thus, the cathodic current participating in the reduction of passive film is too little to reduce the whole film. When there is no CP, the alternating reduction and oxidation of the top surface passive film can consume the AC charge [7]. This top surface passive film is reduced in the negative cycle and re-oxidized in the positive half cycle, leaving the bottom passive film untouched [7], as shown in Figure 12. The whole passive film is nearly intact after the AC cycles. Only a small amount of canary yellow product could be observed on the steel surface and the product increased very little during 120 h immersion under 500 A/m^2^ AC (Figure 4(a1)) as the Fe substrate was protected by the bottom passive film [63] and was not oxidized in the anodic half cycle, as shown by the smooth surface after removing the corrosion products (Figure 4(a2)).

**Figure 13:**
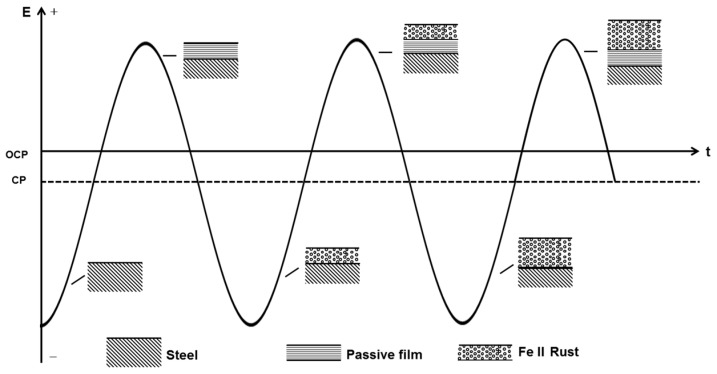
Sketch map of the AC corrosion process occurring on X80 steel in the presence of CP current.

Although without CP only a small part of the cathodic current induced by the negative cycle of AC can reduce the passive film intermittently, the cathodic protection current can cause a continual dissolution of the meta-stable FeCO_3_ passive film. With the application of CP, the pre-cleaning effect removes the previous passive film that forms on the steel surface before AC is applied, and a new redox cycle occurs, including the dissolution of iron, the oxidation of Fe(II), and the reduction of the passive film, as shown in Figure 13. Although the cathodic current involved in the reduction process in the negative cycle only reduces a thin layer of the film, the continuous supply of electrons from the cathodic protection ensures that the entire passive film is reduced into a porous Fe(II) layer. Thus, after the application of an entire cycle of AC in the presence of CP, a non-protective rust layer mainly composed of Fe(OH)_2_ remains on the steel surface, which does not suppress the dissolution of iron substrate in the next anodic cycle so that another layer of Fe(OH)_2_ is deposited over the previous layer after the subsequent AC cycle. Thus, as the oxidation/reduction cycle proceeds, the thickness of the rust layer gradually increases [7].

The long range cyclic voltammetry was from −1.8 to 1.5 V_SCE_, and the positive vertex potential nearly corresponds to the most positive real-time potential of 500 A/m^2^ AC (Figure 7), as shown in Figure 11b. Peak Ia and peak IIa merge with an enhanced current density, which suggests that the increase in AC amplitude accelerates the oxidation rate of iron. Transpassivation occurs at 968 mV_SCE_, which means that pitting may occur when the specimen is exposed to large amplitude AC. However, because the passive film in the pH 9.6 solution is metastable and generally weak, the energy brought by the high voltage is dispersed over the entire film surface, preventing it from breaking the passive film in the absence of CP, as shown in Figure 4(a2). The Fe substrate can remain intact under AC and without CP. However, the corrosion worsens with increasing CP level because the passive film becomes more fragile. This interpretation is confirmed by the surface morphology shown in Figure 4(b1–d1), in which the loose and porous corrosion products were observed on the steel surface under 500 A/m^2^ AC when CP was present. Additionally more corrosion products were observed as the CP potential shifted negatively after 120 h immersion. Therefore, the application of DC potential (CP) is important to the reduction of passive film.

Another effect of the large amplitude AC is the redox of water. The evolution of oxygen that occurs at high positive potential can provide cathodic depolarization agents to promote the reduction reaction. In addition, during the reverse scan, the first cathodic peak appears at a more negative potential of −730 mV_SCE_ with a much stronger current, which translates into enhanced water reduction. A large amount of hydrogen generated by HER can be partly absorbed by or permeate into the iron surface, accelerating the anodic dissolution and destroying the passive film. The effect of hydrogen on the active dissolution kinetics of iron at the potential before the first anodic current peak Ia in Figure 11a can be explained as follows. When the reaction is controlled by the electron transfer step, the internal energy of the metal decreases when it is permeated by hydrogen whereas the entropy increases and the strength of the Fe-Fe bond might be impaired [64], which promotes anodic dissolution. When both the electron-transfer and the film forming-dissolution steps play dominant roles in the potential range between peaks IVa and IIIc, the hydrogen absorbed on the iron surface can react with the passive film through the following reactions:

(2) $${\mathrm{Fe}(\mathrm{OH})}_{2}+{2H}_{\mathrm{ads}}\to {\mathrm{Fe}}^{2+}+{2H}_{2}O+e^{-}$$

(3) $${\mathrm{FeCO}}_{3}+H_{\mathrm{ads}}\to {\mathrm{Fe}}^{2+}+{\mathrm{HCO}}_{3}{}^{-}+e^{-}$$

Therefore, the passive film area decreases. The absorption of hydrogen impedes the film forming process and accelerates film dissolution. Both processes combine with the cathodic polarization to facilitate the generation of the Fe(OH)_2_ reduction-product layer. Thus, as the oxidation/reduction cycle proceeds, the thickness of the rust layer gradually increases until total corrosion finally occurs (Figure 4). These effects can contribute to the phenomenon in which AC corrosion often becomes more severe as the amplitudes or current densities increase.

### 3.4. Analysis of Raman Spectroscopy

**Figure 14:**
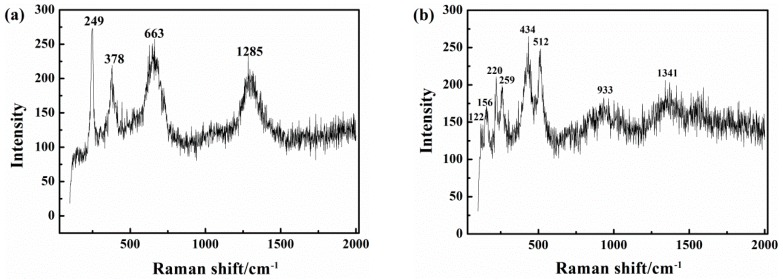
Raman spectra of the corrosion products on the X80 steel after 120 h immersion in pH 9.6 solution under i_AC_ 500 A/m^2^ and various CP: (**a**) CP = 0; (**b**) CP = −1000 mV_SCE_.

Figure 14 shows the results of Raman spectra analysis of the corrosion products. In the pH 9.6 solution, in the absence of CP (Figure 14a), the 663 cm^−1^ peak of Fe_3_O_4_ is intense, as are the 249 and 378 cm^−1^ peaks of γFeOOH. Both oxidation products are unstable [57] and can be attributed to Equation (4).

(4) $$6{\mathrm{FeCO}}_{3}+O_{2}+{6H}_{2}O\to {2\mathrm{Fe}}_{3}O_{4}+{6\mathrm{HCO}}_{3}{}^{-}+{\mathrm{OH}}^{-}$$

In the presence of CP at −1000 mV_SCE_ (Figure 14b and Table 2), except for the three small peaks of αFe_2_O_3_ below 250 cm^−1^, that are due to αFe_2_O_3_ and might be generated by secondary oxidation in air of the original corrosion products, the 434 cm^−1^ peaks of Fe(OH)_2_ demonstrate the reducing effect of cathodic protection current on the passive film.

### 3.5. Interface Properties

**Figure 15:**
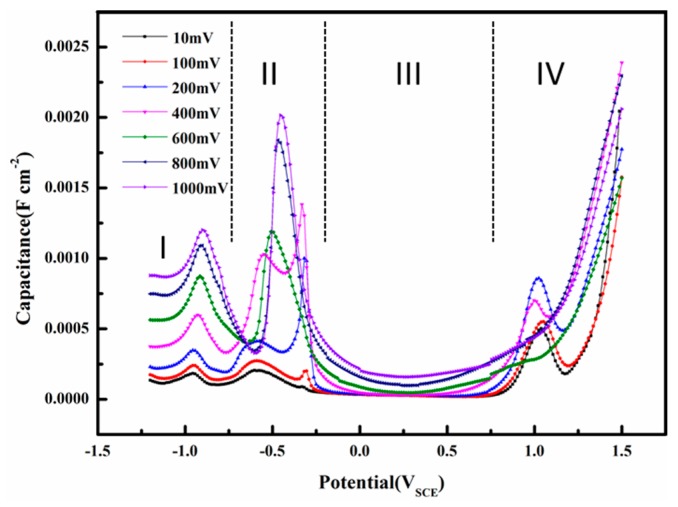
Capacitances of the X80 steels in pH 9.6 alkaline solution under 50 Hz AC with various amplitudes. (**I**) a double layer region; (**II**) an oxidation region; (**III**) a passive region; and (**IV**) a transpassive region.

The interfacial capacitance is calculated using Equation (1). This capacitance is different from conventional capacitances by definition, but it can be used for analysis of the interface properties in AC corrosion [15] or investigation of double layer capacitance with large amplitude sinusoidal voltammetry [16,17]. The capacitances were used to evaluate the electrode/solution interface properties of X80 steel in a pH 9.6 alkaline environment. Figure 15 shows the influence of AC voltage amplitude on the capacitance as a function of DC potential. The interfacial capacitance consisted of a series combination of the double layer and oxide capacitances [25]. The composition of oxide films and the nature of the double layer are closely related to the capacitance [65]. In the pH 9.6 solution, as shown in Figure 15, four regions are apparent in the capacitance vs. DC potential curves: (I) a double layer region; (II) an oxidation region; (III) a passive region; and (IV) a transpassive region.

**Figure 16:**
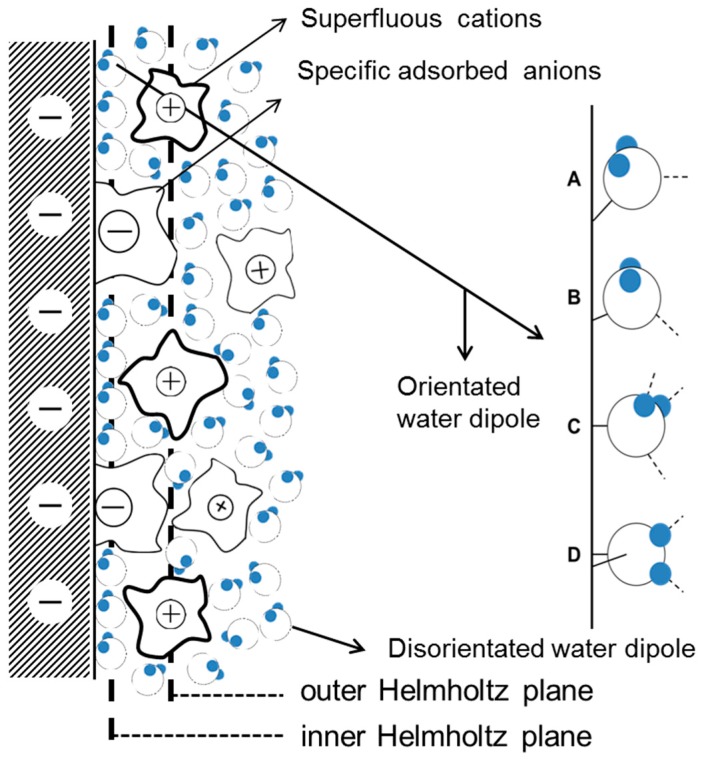
Schematic representation of the influence of alternating electric field on the orientation of water dipoles in the double layer: (**A**) when potential is below potential of zero charge (pzc); (**B**) around the pzc; (**C**) slightly above the pzc; (**D**) more positive potentials.

In region I, when AC is small, the electrode can be largely protected by the DC cathodic current because the effect of DC potential on corrosion rates predominates when the DC potential is out of the Tafel region [15,26]. Therefore, assuming that the capacitance of the corrosion product layer was neglected, the interfacial capacitance can be approximately regarded as the single double layer capacitance. The capacitance in the double layer region increases with the AC amplitude, which may be related to the AC induced changes in water molecules adsorbed on the surface. The widely accepted double layer model proposed by Stern is illustrated in Figure 16.

As seen from Figure 16, the adsorption of water on metal surfaces is an essential part of the double layer structure and electrochemical reactions. The integration capacitance can be calculated using Equation (5) below:

(5) $$C=\frac{\varepsilon_{r}\varepsilon_{0}}{L}$$

where C (F/m^2^) is the integration capacitance of the double layer, $\varepsilon_{0}$(F/m) is the vacuum dielectric constant, $\varepsilon_{r}$ is the dielectric constant of the water, and L (m) is the distance between two parallel plates.

The dielectric constant of the water is greatly influenced by the orientation of the water molecules. At 25 °C the water molecule dipole can rotate freely under the influence of an electric field, ε_r_ = 80. The orientation of water molecules is restricted when they are between the inner Helmholtz plane and outer Helmholtz plane, and ε_r_ = 30. When the water molecules are influenced by the electric field and by a chemical force in the inner Helmholtz layer, their orientation is determined a priori by the charge on the metal surface and ε_r_ = 6. Therefore, the dielectric constant of water increases with the orientation freedom of the water molecule dipoles. As assumed by the classical models of electrochemical interfaces [66], the orderly arranged water molecules in the inner Helmholtz layer can reorient from “oxygen-up” to “oxygen-down” as the electrode potential changes from negative to positive [67,68]. In the double layer region, the application of 50 Hz AC causes the quick charge-discharge cycle of the metal surface, and alternating swings of the electrode potential between negative and positive occur as the AC amplitude increases. Thus, the orientation of water molecules continually changes and the dielectric constant of the water will correspondingly increase, leading to the increase of capacitance (Equation (5)). The long-range electrostatic adsorption of anions and cations are greatly weakened by the charge-discharge effect. Consequently, the absorption of neutral water molecules is relatively strengthened, which can also contribute to the increase of capacitance because the dielectric constant of the water is greater than that of most ions. In addition, the wave bottom at −800 mV_SCE_ is supposed to be related to the electrode potential of zero charge (pzc) because the local density of interfacial water is minimal around pzc.

In region II, when loose and porous corrosion products form on the surface of the electrode, the surface can be regarded as the surface of a porous electrode [69]. The interfacial capacitance is extremely large (regions II and IV in Figure 15) and the corrosion product layer contributes much more to this phenomenon than the double layer. Therefore, assuming that the double layer capacitance was neglected, the interfacial capacitance can be approximately regarded as the single corrosion product layer capacitance. Peaks II and III merge into a single earlier peak as the AC amplitude increases above 400 mV_rms_, which means that the extra anodic current brought by AC can promote oxidation of Fe.

In region III, a passive film is formed in this DC potential range (Figure 11). The interfacial capacitance is very small and it can be attributed to the space charge capacitance of the passive film [69]. Therefore, assuming that the double layer capacitance was neglected, the interfacial capacitance can be approximately regarded as the space layer capacitance of the passive film. The capacitances remain unchanged from AC 10 to 400 mV_rms_, but begin to rise as the AC amplitude increases from 600 to 1000 mV_rms_, indicating that the redox reaction during the application of a large amplitude AC repeatedly attacks and restores the passive film so that a rough and porous surface forms at the passive film-electrolyte interface. The increase of capacitances can be attributed to the additional surface area [70]. When the AC amplitudes are small enough, i.e., 10 to 400 mV_rms_, to permanently maintain the peak and bottom electrode potentials within the passive region, the passive film will remain protective and the capacitances remain the same.

In the transpassive region IV, the film under the small amplitude AC suffers from a sudden attack after the DC potential becomes greater than the pitting potential, resulting in a sharp increase of capacitance [71]. However, the capacitance of the passivation film influenced by large amplitude AC, which has already been attacked by AC during the passive region, increases steadily, showing no sign of sudden failure.

## 4. Conclusions

The X80 steel under the combined impact of AC and CP current presents different corrosion behaviors in non-passive and passive systems containing bicarbonate ions. The following conclusions can be deduced from our results:

(1) In the non-passive system with pH 7.2, AC could change the CP potential from the original value and the shifting direction of the CP potential depends on the applied cathodic potential. The minimum CP criterion −840 mV_SCE_ cannot fully protect the steel in the presence of AC, but the steel can remain intact in the presence of i_AC_ 100 A/m^2^ (Vp^+^ 2.69 V_SCE_ and Vp^−^ −4.34 V_SCE_) when the CP potential decreases negatively enough, such as −1000 mV_SCE_ and −1200 mV_SCE_.
(2) In the alkaline environment with pH 9.6, AC corrosion is negligible without CP, but accelerates dramatically as the CP potential becomes more negative when i_AC_ is 500 A/m^2^ (Vp^+^ 1.63 V_SCE_ and Vp^−^ −3.39 V_SCE_).
(3) The dielectric constant of water molecules absorbed on the electrode surface in the inner Helmholtz layer increases with the increase of AC voltage amplitude, resulting in the increase of interfacial capacitance between the electrode and solution.

## Figures and Tables

**Table 1:** Chemical compositions of X80 (wt. %).

Material	Fe	C	Mn	Si	S	Cr	Ni	Cu	Al	Ti	Mo	V	Nb	N
**X80**	96.933	0.036	1.771	0.197	0.002	0.223	0.278	0.22	0.021	0.019	0.184	0.001	0.11	0.005

**Table 2:** Raman shift and the corresponding chemical species in two different cathodic protection (CP) conditions.

Test Condition	Raman Shift (Wave number/cm^−1^)	Species
pH 9.6 AC = 500 A/m^2^ CP = 0	249, 378, 1285	γFeOOH
	663	Fe_3_O_4_
pH 9.6 AC = 500 A/m^2^ CP = −1000 mV_SCE_	122, 155, 220	γFe_2_O_3_
	259	γFeOOH
	434	Fe(OH)_2_
	512	γFe_2_O_3_
	1341	Fe(OH)_3_
